# Calcifying Bacteria Flexibility in Induction of CaCO_3_ Mineralization

**DOI:** 10.3390/life10120317

**Published:** 2020-11-28

**Authors:** Darya A. Golovkina, Elena V. Zhurishkina, Lyubov A. Ivanova, Alexander E. Baranchikov, Alexey Y. Sokolov, Kirill S. Bobrov, Alexey E. Masharsky, Natalia V. Tsvigun, Gennady P. Kopitsa, Anna A. Kulminskaya

**Affiliations:** 1 Petersburg Nuclear Physics Institute Named by B.P. Konstantinov of National Research Centre “Kurchatov Institute”, 188300 Gatchina, Russia; darya_golovkina@mail.ru (D.A.G.); zhurishkina_ev@pnpi.nrcki.ru (E.V.Z.); luba_1305@inbox.ru (L.A.I.); aleksoko@gmail.com (A.Y.S.); bobrov_ks@pnpi.nrcki.ru (K.S.B.); kopitsa_gp@pnpi.nrcki.ru (G.P.K.); 2 Kurchatov Genome Centre-PNPI, 188300 Gatchina, Russia; 3 Kurnakov Institute of General and Inorganic Chemistry of the Russian Academy of Sciences, 119991 Moscow, Russia; a.baranchikov@yandex.ru; 4 Core Facility Centre for Molecular and Cell Technologies, St. Petersburg State University, 198504 St. Petersburg, Russia; alexey.masharsky@spbu.ru; 5 Federal Scientific Research Centre “Crystallography and Photonics”, Russian Academy of Sciences, 119333 Moscow, Russia; n_tsvigun@mail.ru

**Keywords:** calcium carbonate, biomineralization, ureolytic bacteria, polymorph

## Abstract

Microbially induced CaCO_3_ precipitation (MICP) is considered as an alternative green technology for cement self-healing and a basis for the development of new biomaterials. However, some issues about the role of bacteria in the induction of biogenic CaCO_3_ crystal nucleation, growth and aggregation are still debatable. Our aims were to screen for ureolytic calcifying microorganisms and analyze their MICP abilities during their growth in urea-supplemented and urea-deficient media. Nine candidates showed a high level of urease specific activity, and a sharp increase in the urea-containing medium pH resulted in efficient CaCO_3_ biomineralization. In the urea-deficient medium, all ureolytic bacteria also induced CaCO_3_ precipitation although at lower pH values. Five strains (*B. licheniformis* DSMZ 8782, *B. cereus* 4b, *S. epidermidis* 4a, *M. luteus* BS52, *M. luteus* 6) were found to completely repair micro-cracks in the cement samples. Detailed studies of the most promising strain *B. licheniformis* DSMZ 8782 revealed a slower rate of the polymorph transformation in the urea-deficient medium than in urea-containing one. We suppose that a ureolytic microorganism retains its ability to induce CaCO_3_ biomineralization regardless the origin of carbonate ions in a cell environment by switching between mechanisms of urea-degradation and metabolism of calcium organic salts.

## 1. Introduction

Biomineralization is known as a process of induction of mineral formation by living organisms within their metabolic reactions with the environment. To date, formations of calcium carbonate crystals of various morphology have been reported to be induced by different bacterial strains. In addition to numerous representatives of *Bacillus*, *Sporosarcina pasteurii* [1,2], *Synechococcus* sp. as well as species of *Arthrobacter crystallopoietes*, *Rhodococcus qingshengii*, *Psychrobacillus psychrodurans* [3,4] are known to induce calcium carbonate crystallization. The most common polymorph is calcite that was formed during the growth of *B. megaterium*, *B. licheniformis*, *B. flexus* and *B. pasteurii* strains [5,6,7]. *B. sphaericus* have been shown to induce crystallization of various types of carbonates under alkaline conditions and at low temperatures [8]. Notably, only the fungal strain of *Aspergillus nidulans* capable of growing on concrete slabs [9] was shown to induce calcium carbonate mineralization.

During the last decade, an alternative innovative biotechnological approach based on microbial systems providing CaCO_3_ crystallization inside of microcracks on the surface of concrete has been actively developing [10,11,12,13]. Microbially induced precipitation seems to be an environmentally friendly process [14] and has the potential to launch inside the cracks spontaneously [7,10,11,12,13,15,16]. Beside the attempts to develop an effective technology for cement self-healing using biomineralization, biogenic CaCO_3_ is being used to create new biomaterials based on different polymorphs of the mineral [17,18].

**Figure 1:**
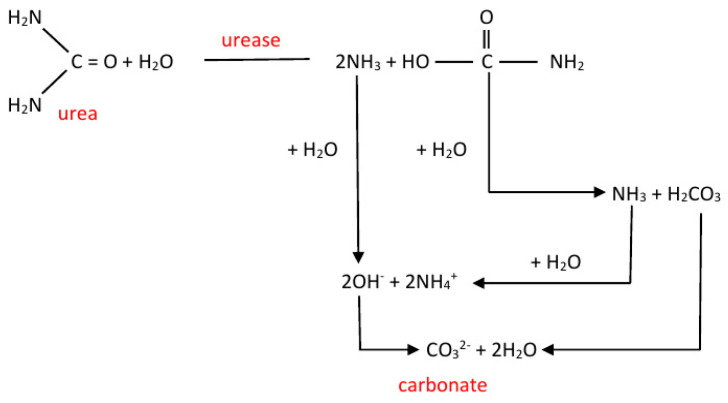
Schematic illustration of urease-driven calcium carbonate precipitation induced by an ureolytic microorganism.

The induction of CaCO_3_ precipitation by microorganisms is known to be able to accompany two fundamentally different metabolic routes: heterotrophic and autotrophic. Three mechanisms are distinguished in autotrophy: non-methylotrophic methanogenesis, oxygenic and anoxygenic photosynthesis [19,20]. In all three pathways, CO_2_ is used as a carbon source for the production of organic compounds yielding in a local deficit of CO_2_ in the medium or the bacterial cell environment and, as a consequence, the induction of CaCO_3_ precipitation in the presence of Ca^2+^ ions near the cell. In the heterotrophic pathway, organic compounds are used as a source of energy following the oxidation of sulfur compounds, or organic acid salts or the nitrogen cycle as the main mechanism. In turn, the nitrogen cycle includes the ammonification of amino acids, the dissimilatory decomposition of nitrates, and the urea degradation pathway [21]. The most studied and easily detectable laboratory process of microbial-based induction of CaCO_3_ precipitation is the production of large amounts of carbonates during urea degradation by ureolytic bacteria [22,23,24] and many others. Urease (or ureaaminohydrolase, E.C. 3.5.1.5) catalyzes the hydrolysis of urea to carbon dioxide and ammonia. The appearance of NH_4_^+^ ions in the reaction medium increases its pH rapidly. Carbon dioxide combines with water to form carbonic acid, which then dissociates yielding carbonate ions (Figure 1).

**Figure 2:**
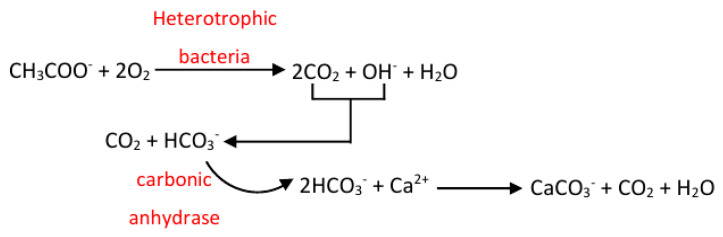
Chemical reactions leading to CaCO_3_ crystallization through the oxidation of Ca acetate salt induced by heterotrophic bacteria.

The less studied mechanism of induction of calcium carbonate crystallization through the oxidation of Ca organic acid salts represents an alternative to the urease-aided method. This mechanism is based on the oxidation of the organic component to CO_2_ and OH^−^ ions catalyzed by microbial carbonic anhydrases (Figure 2). As a result of the medium alkalization due to the presence of hydroxyl ions, CO_2_ is transformed into carbonic acid followed by conversion into HCO_3_^−^ and H^+^ ions. Furthermore, in the presence of free calcium ions in the medium, calcium carbonate is formed [25].

Our initial goals were to find microorganisms capable of inducing mineral formation among cultures from our collection and to identify candidates for the development of technology for autogenous crack healing in cement samples. To address the requirements applied to microorganisms that could be used as concrete self-healing agents, the screening strategy included a search for Gram-positive ureolytic bacteria capable of surviving in alkaline conditions. We were focusing on ureolytic bacteria since they are the most studied and widely applied for such technologies. However, for practical purposes, the use of urease-driven biomineralization is not always suitable due to a negative effect of the by-products formed during urea degradation and some other disadvantages like the temperature dependence of the enzyme catalytic activity, which influences the process efficiency [24]. Therefore, more efficient and environmentally friendly methods of using microorganisms in the processes of autogenous healing of cementitous structures are needed. The reported effect of both ureases and carbonic anhydrases on microbially induced CaCO_3_ precipitation (MICP) [26,27] prompted us to compare the kinetics of calcite precipitation by the selected ureolytic bacteria in media with and without urea. As a result, our research has provided new insights into the understanding of the processes that underlie MICP. This could lead to the development of tools to control the course of biomineralization in order to produce certain CaCO_3_ polymorphs required for the creation of new biomaterials.

## 2. Materials and Methods

### 2.1. Bacterial Strains, Screening and Cultivation Conditions

Bacterial strains used in the study were from the in-house collection of the Enzymology laboratory of B.P. Konstantinov Petersburg Nuclear Physics Institute NRC “Kurchatov Institute” and were maintained on LB agar medium (peptone 10 g/L, yeast extract 5 g/L and NaCl 10 g/L). For screening purposes, bacterial stains were transferred onto LB-agar with pH 9 (adjusted with 5 M NaOH solution) and grown for 3 days at 37 °C. Gram-positive cultures capable of growing on an alkaline medium were then transferred to starvation agar (30 g agar and 1 L of distilled water) and cultured for 2 days at 37 °C followed by the selection of bacteria that formed a large number of endospores observed microscopically.

For assessment of the ability of selected bacterial cultures to induce calcium carbonate precipitation, each bacterial strain was grown in 10 mL of medium YPG containing 0.5% (*w*/*v*) peptone, 0.5% glucose, 0.05% yeast extract [28] for one day at a temperature of 37 °C with shaking (at 100 rpm). Then the tube with each culture was heated at 80 °C for 10 min and cells were separated by centrifugation at 2000× *g* for 10 min. The precipitate was suspended in 2 mL of 0.9% NaCl solution and transferred into 100 mL of liquid medium B4-AC or B4-U. Medium B4-AC contained: calcium acetate, 2.5 g/L; yeast extract, 2 g/L; glucose, 10 g/L, pH 7.2. Medium B4-U contained: yeast extract, 2 g/L; glucose, 10 g/L; urea, 2.5 g/L; CaCl_2_, 2.5 g/L, pH 5.3. Molar calcium concentrations were approximately the same in each medium (1.6 mM in B4-AC and 2.2 mM in B4-U). Pre-sterilized urea, CaCl_2_ or Ca acetate solutions were added separately to the medium after autoclaving. Bacteria were cultivated for 14 days at 37 °C with shaking at 110 rpm. The resulted calcium carbonate precipitates were collected on a filter membrane with a pore size of 0.2 μm, washed two times with distilled water to remove cells and culture medium, oven dried at 50 °C for 48 h and sent for analysis.

For each selected bacterium, changes in cultivation medium pH and urease activity, biomass accumulation and precipitation yields during 14-day growth in both media were monitored. A sample without bacteria was used as a chemical control and *E. coli* DH5α was used as a negative control. Periodically withdrawn 5-mL aliquots were cooled to the room temperature followed by measurements of pH, optical density at 595 nm and urease activity. In each sample, insoluble precipitates were treated as above and weighed.

To evaluate the mineralizing ability of the strain *B. licheniformis* DSMZ 8782, the inoculate was transferred into the flasks with 100 mL of the liquid medium B4-U or B4-AC and cultivated for 14 days at 37 °C with shaking at 110 rpm with periodic removing of 5-mL aliquots. Withdrawn aliquots were cooled to the room temperature followed by measurements of pH, optical density at 595 nm and urease activity. In each sample, insoluble precipitates were treated as above and analyzed.

### 2.2. Strain Identification

Genomic DNA was isolated from the overnight bacterial cultures using GeneJET Genomic DNA Purification Kit (Thermo Fisher Scientific Baltics UAB, Vilnius, Lithuania) according to the provided protocol. The PCR was carried out with Taq DNA polymerase (Evrogen, Moscow, Russia) and universal primers 8F and 1492R (l) for the 16S rRNA gene [29] in the Eppendorf Mastercycle Personal. The agarose gel electrophoresis confirmed the formation of only one product with the length about 1500 bases for all strains (data not shown). The PCR products were purified with the phenol-chloroform extraction [30] and sequenced. The sequencing was performed at the Core Facility Center for Molecular and Cell Technologies. Resulting sequences were aligned against the database of 16S ribosomal RNA sequences using the program BLASTN 2.9.0+ [31]. Multiple sequence alignment by ClustalW and phylogenetic analysis were performed using the MEGA X (version 10.1.8) software.

### 2.3. Nucleotide Sequence Accession Numbers

The nucleotide sequence data reported are available in the GenBank database under the accession number(s): MW251744 (*Micrococcus luteus* 6), MW251742 (*B. cereus* 4b), MW251741 (*Staphylococcus epidermidis* 4a); MW251743 (*B. cereus* 168), MW251746 (*M. luteus* BS52), MW251745 (*B. cereus* BSP), MW251747 (*B. subtilis* K51), and MW251748 (*B. subtillus* 170).

### 2.4. Urease Assay

Urease activity was determined according to the method described in [28] by evaluation of the conductivity of a culture medium. To assess the urease activity, bacterial strains were grown in 5 mL of beef broth containing 3 g/L of beef extract and 5 g/L of peptone for 24 h at 37 °C with shaking (100 rpm). The resulting bacterial cells were separated by centrifugation at 3000 rpm for 5 min. The precipitate was suspended in 0.9% NaCl solution, adjusted to optical density 0.5 at 600 nm, and 0.5 mL of the suspension was added to 40 mL of B4-U medium. The medium did not contain CaCl_2_ to avoid CaCO_3_ crystal formation that makes the conductivity assay difficult. Bacteria were cultivated for 14 days at 37 °C with shaking (100 rpm) and aliquots (5 mL) were withdrawn daily for pH and urease assays. For the latter, the aliquot was cooled to 25 °C and the conductivity was measured using a conductometer DFRobot DFR0300-H Gravity: Analog Electrical Conductivity Sensor/Meter (K = 10). The formation of ionic particles of non-ionic substrates led to an increase in the total conductivity of the solution and the rate at which the conductivity increased was proportional to the concentration of the active urease presented in the reaction mixture. The conductivity of the medium with growing *E. coli* DH5α strain was taken as a negative control. Molar concentration of degraded urea was calculated using the Equation (1) with the coefficient taken from [32]:

Ureahydrolysed (mM) = Conductivity (mS) × 11.11(1)

One unit of urease activity corresponded to the enzyme amount capable of catalyzing the conversion of 1 μM urea per minute under the standard assay conditions (pH 5.5, 37 °C, 20 min). The data points are presented as the means of at least three independent experiments, and the errors were calculated for each data point using Excel Solver add-in (Microsoft, Redmond WA, USA). The time-dependent curves reported below for pH- and urease activity assays during the bacterial growth were generated with ORIGIN 8.0 software (OriginLab, Northampton, MA, USA).

### 2.5. XRD and SEM Analysis

Powder X-ray diffraction (XRD) analysis of all samples was performed on a Bruker D8 Advance diffractometer (Bragg-Brentano geometry) with Ni-filtered CuKα (λ = 1.5418 Å) radiation and a LYNXEYE detector. Diffraction patterns were recorded in the 10–70° 2θ range, with a step of 0.02° and collection time of 0.3 s/step. Rietveld analysis of the patterns was carried out with a help of FullProf Suite [33]. Structure models were obtained from Crystallography Open Database [34].

The microstructure of the samples was investigated using a Carl Zeiss NVision 40 high resolution scanning electron microscope at 1 kV acceleration voltage.

### 2.6. Restoration of Concrete Cracks with Selected Bacteria

Dry grade M300 non-sterilized cement was diluted in distilled water and poured into silicone molds. After the cement had completely solidified, the samples (Ø 4.5 cm, h 1.0 cm) were removed and micro-cracks were made using a marble pestle. Each selected bacterial strain was grown in 10 mL of YPG medium for one day and centrifuged at 2000× *g* for 10 min. Cells were re-suspended in 5 mL of 0.9% NaCl and sterile transferred to 100 mL of the medium B4-U. Then 30 mL of each bacterial suspension were applied to a cement sample with cracks. Cement samples were put at 37 °C with periodical addition of the nutrient medium. A sample without the addition of bacteria was taken as a control. After a month of observation, the samples were dried and visually analyzed using a MSP-1 optical microscope.

## 3. Results and Discussion

### 3.1. Screening and Characterization of CaCO_3_ Mineralyzing Bacteria

A laboratory microbial collection containing 71 bacterial strains that have been gathered from various locations and purchased from different microbial collections were used to screen for ability to induce CaCO_3_ precipitation. Considering the requirements applied to microorganisms that could be used as concrete self-healing agents, we used a specific screening strategy. At the first stage, bacteria able to survive in alkaline conditions were selected. This stage was necessary because freshly prepared concrete has a pH value in the range of 8 to 11. As a result, 28 strains capable of growing on LB medium with a pH 9 were found among 71 strains taken into the study. The next task was to determine Gram-positive bacteria as potentially able to form spores, since the strains should retain their viability for several years to be used as a restoring material in the place of formation of the opening crack. As a result, 9 cultures out of 13 Gram-positive bacterial strains appeared to be able to withstand starvation growth conditions including six strains with the ability to produce abundant endospores that were determined microscopically.

**Figure 3:**
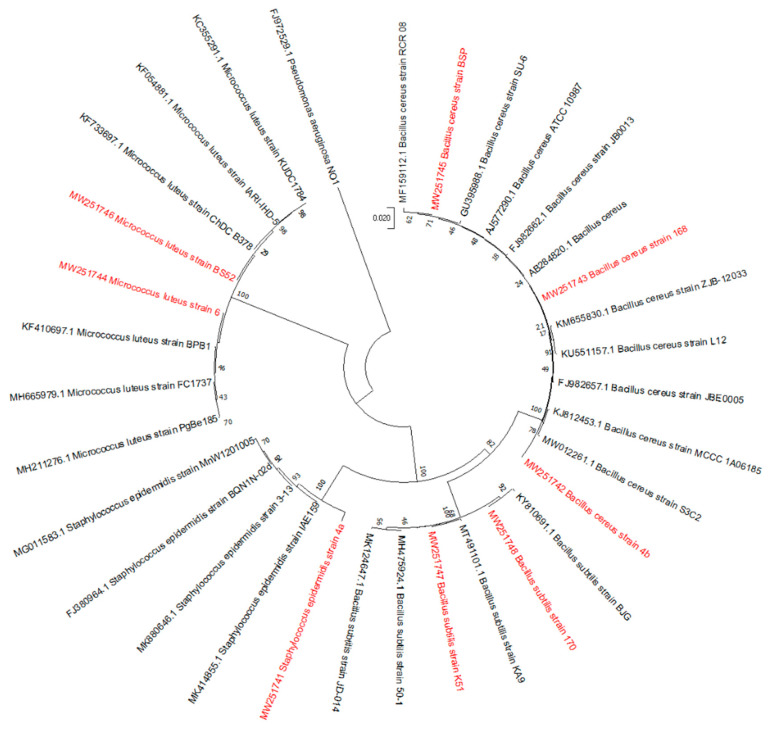
Phylogenetic tree constructed with the neighbor-joining method from partial 16S rDNA gene sequences of the eight isolates and reference strains (accession numbers are indicated). Species used as the outgroup was *Pseudomonas aeruginosa*. Bootstrap probabilities as determined for 1000 replicates are given as a percentage.

A 16S rRNa gene of eight earlier non-identified microorganisms were partially sequenced and the phylogenetic analysis of these sequences was performed (Figure 3), whereby these microorganisms were identified as: *Micrococcus luteus* 6, *Bacillus cereus* 4b, *Staphylococcus epidermidis* 4a; *B. cereus* 168, *M. luteus* BS52, *B. cereus* BSP, *B. subtilis* K51, and *B. subtillus* 170. Three strains (*S. epidermidis* 4a, *M. luteus* 6 and *M. luteus* BS52) did not produce endospores but due to the production of cyst-like complexes during the starvation stage they were included into the list of studied microorganisms.

**Figure 4:**
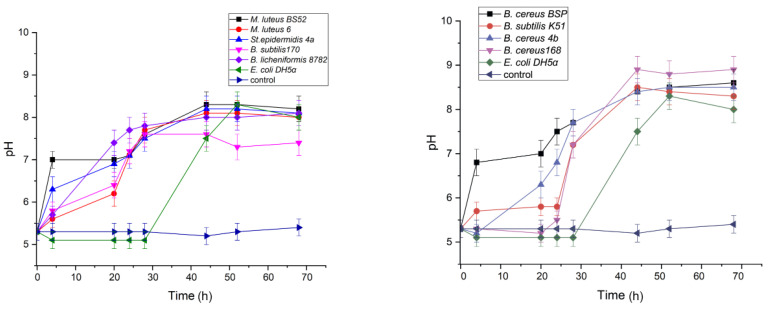
Time-dependence of the pH values during the bacteria growth in B4-U medium. Blue line represents data for a sample without bacteria used as a chemical control and green line represents data for *E. coli* DH5α used as a negative control. Data points are presented as the means of three independent experiments, the errors were calculated for each data point using Excel Solver add-in (Microsoft, Redmond, WA, USA).

The ability of the selected cultures to induce precipitation of calcium carbonate was monitored during their growth in a liquid urea-supplemented medium B4-U. The values for the medium pH have been increasing from 5 to 7–8.5 during the first two days for all cultures and remained constant until the end of the experiment (Figure 4).

**Figure 5:**
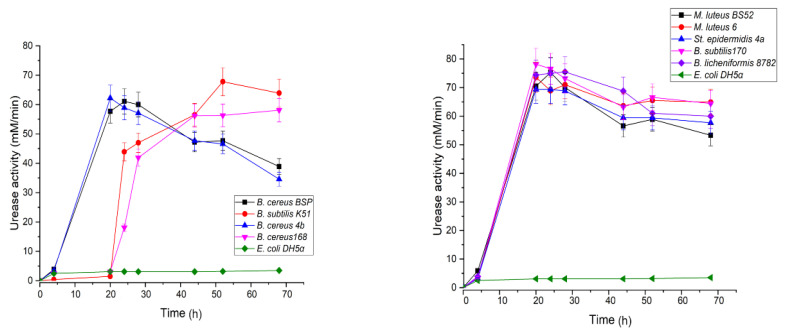
Time-dependence of the specific urease activity of each tested strain during the growth in the B4-U medium. The specific urease activity was assessed using measured value for conductivity of the reaction mixture according to the Formula (1). Green line represents data for *E. coli* DH5α used as a negative control. The data points are presented as the means of three independent experiments, and the errors were calculated for each data point as described for Figure 2.

Analysis of the time-dependent curves of the specific urease activity for each strain revealed two types of behavior: the majority of the strains showed the maximal activity after the first day of cultivation following by some decrease during the next two days, whereas urease activity of *B. subtilis* K51 and *B. cereus* 168 reached maximum after 44–50 h and then remained constant (Figure 5).

All selected ureolytic strains appeared to induce calcium carbonate mineralization in the urea-deficient medium (B4-AC) showing almost the same precipitate yields as in the medium with urea B4-U (see Table 1). We observed that after 14 days of cultivation the highest amount of CaCO_3_ in B4-U medium was produced by the strains *M. luteus* 6, *S. epidermidis* 4a, *B. cereus* 4b while in the B4-AC medium *M. luteus* BS52 and *B. cereus* BSP showed the best results (Table 1).

### 3.2. CaCO_3_ Mineral Analysis

All samples of bacterial CaCO_3_ precipitates obtained after the two-week growth of selected strains in B4-U and B4-AC media were analyzed by powder X-ray diffraction (XRD) spectrometry using the Rietveld method. Some samples were monophase but the majority represented the mixture of calcite [35], vaterite [36] and non-identified phase in various combinations (Figure S1a,b, in the Supplementary Materials). Neither aragonite [37], nor hydrated forms (monohydrocalcite and ikaite) [38] were detected. Here, calcite formation was recorded in six of nine studied strains after their growth in urea-containing medium B4-U (Table 2).

Vaterite, in addition to calcite, was detected for *B. subtilis* 170 and *B. cereus* 168 grown in B4-U medium and for all strains except *B. subtilis* K51 and *B. licheniformis* DSMZ 8782 during their growth in B4-AC. Surprising was the appearance of an unidentified phase in some samples (Figure S1c).

We found only one recent report by Ghosh with co-authors [39] where an unidentified phase was mentioned with respect to the bacterium *Sporosarcina pasteurii* biomineralization. Most probably, the appearance of peaks corresponding to the unidentified phase may indicate the appearance of a crystalline substance due to the interaction of calcium ions with organic species present in the culture media. It was observed for four strains grown in B4-U medium and for seven strains grown in calcium acetate medium B4-AC (Table 2). In the B4-AC medium, calcite was formed by all microorganisms except *M. luteus* BS52, for which only vaterite and the unidentified phase were detected. It may indicate that thermodynamically unstable ACC and metastable vaterite phases have not been transformed into stable calcite by the end of the *M. luteus* BS52 growth in urea-deficient medium. For the strains *S. epidermidis* 4a and *B. cereus* 4b calcite and vaterite were detected in B4-AC medium and only the unidentified phase was observed in B4-U medium. SEM analysis of the precipitates produced by the selected strains after a 2-week growth in both media also confirmed that the samples consisted of strongly aggregated particles having various shapes (Figure S2, Supplementary Materials).

**Figure 6:**
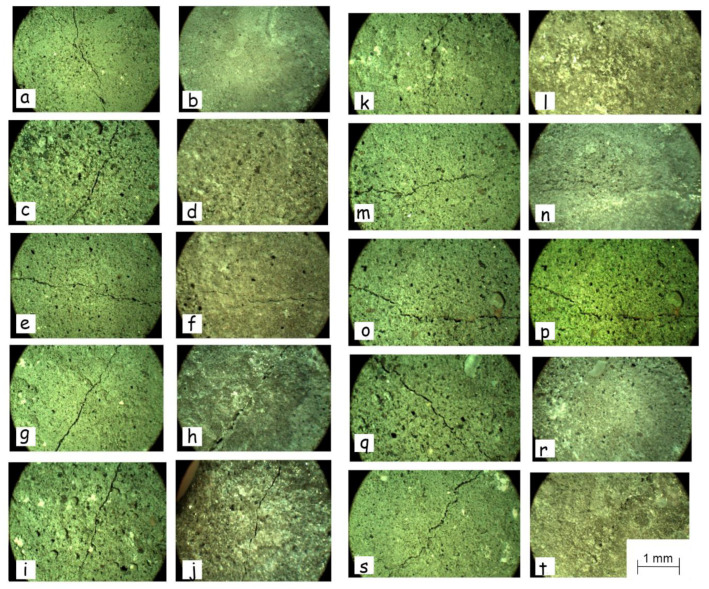
Typical micrographs of microcrack filling by the selected strains before and after a month of the growth on the cement surface: *B. licheniformis* DSMZ 8782 (**a**,**b**); *B. cereus* 4b (**c**,**d**); *B. cereus* 168 (**e**,**f**); *B. cereus* BSP (**g**,**h**); *E. coli* DH5α, negative control (**i**,**j**); *S. epidermidis* 4a (**k**,**l**); *M. luteus* 6 (**m**,**n**); *B. subtillus* 170 (**o**,**p**); *M. luteus* BS52 (**q**,**r**); *B. subtilis* K51 (**s**,**t**). Cement samples were 4.5 × 1 cm in size; microcracks were 3.5–4 cm in length and 0.12–0.4 mm in width).

To evaluate the potential of the selected strains for practical applications, we have tested them in cement-sand microcracks healing. It should be taken into account that the microcracks in the cement-sand samples were made manually, so their size and distribution could also affect the rate of the crack repair process. Since we used this method only for screening purposes, we have been considering the results after a month of treatment by bacteria to exclude the influence of the difference in the lesions size on healing. Thus, the selected strains demonstrated different abilities to restore micro-cracks on the cement-sand surface. The strains *B. licheniformis* DSMZ 8782 (Figure 6a,b), *B. cereus* 4b (Figure 6c,d), *S. epidermidis* 4a (Figure 6k,l), *M. luteus* BS52 (Figure 6q,r), and *M. luteus* 6 (Figure 6m,n) appeared to repair microcracks in the samples while the effect of cultivation of *B. cereus* 168 (Figure 6e,f), *B. cereus* BSP (Figure 6g,h), *B. subtilis* 170 (Figure 6o,p) and *B. subtilis* K51 (Figure 6s,t) was negligible. *E. coli* DH5α, negative control (Figure 6i,j).

### 3.3. Analysis of Precipitation Process Induced by B. licheniformis DSMZ 8782

**Figure 7:**
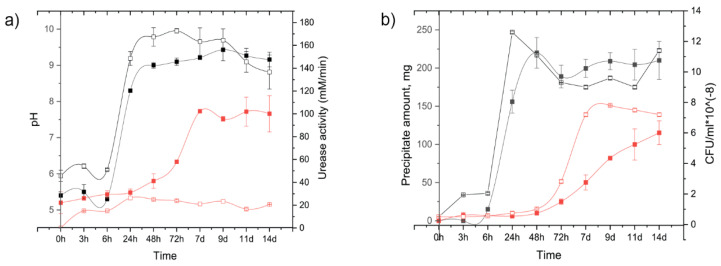
Time-dependences of *B. licheniformis* growth parameters in 100 mL of B4-U medium (black lines) and B4-AC medium (red lines): (**a**) pH (filled squares) and specific urease activity (open squares); (**b**) CaCO_3_ precipitate amount (filled squares) and bacterial biomass accumulation (open squares). Errors bars represent average and standard deviation of three replicates.

The strain *B. licheniformis* DSMZ 8782 was the fastest in microcracks healing. Considering the observation that it produced only the most stable polymorph, calcite, during its growth in liquid media, this strain was further studied in more details. During a two-weeks cultivation of the *B. licheniformis* DSMZ 8782 in liquid B4-U and B4-AC media with sequential aliquot sampling at regular intervals, the changes in the growth media pH values, biomass accumulation, CaCO_3_ precipitation, and the specific urease activity were monitored (Figure 7).

Figure 7 shows that during the growth in B4-U medium, pH values increased rapidly after the first day and then remained constant; the exponential growth phase of the bacterium corresponded to the intense crystal formation and a linear increase in the urease rate. As expected, no urease activity was detected in the B4-AC medium, indicating that crystal formation is proceeded by a different mechanism.

Finally, cell suspensions of *B. licheniformis* DSMZ 8782 were applied to completely dried cement-sand samples. A month later, the sample treated with the bacterium in B4-U medium was completely repaired, while in the case of using the B4-AC medium, the crack was not closed up totally but a white coating by calcium carbonate appeared (Figure S3, Supplementary Materials). This agrees with our results obtained during the bacterial growth in liquid media (Figure 7). When using the urea-rich medium, urease was induced and, accordingly, an intense CaCO_3_ precipitation occurred. Conversely, in the B4-AC medium, the peak of precipitate production was reached much later, at the beginning of the second week.

**Figure 8:**
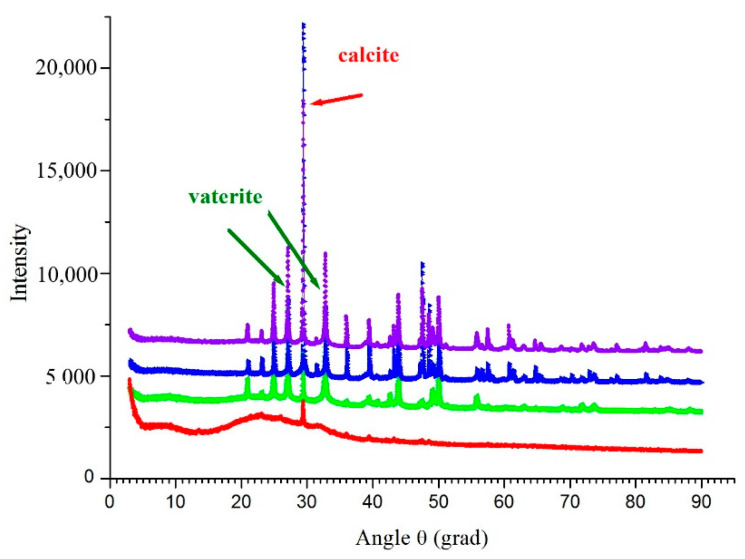
Typical X-ray diffraction patterns of CaCO_3_ precipitates formed by the strain *B. licheniformis* DSMZ 8287 during 14-days growth: after 29 h, red line; 72 h, green line; 9 days, blue line; 14 days, violet line.

**Figure 9:**
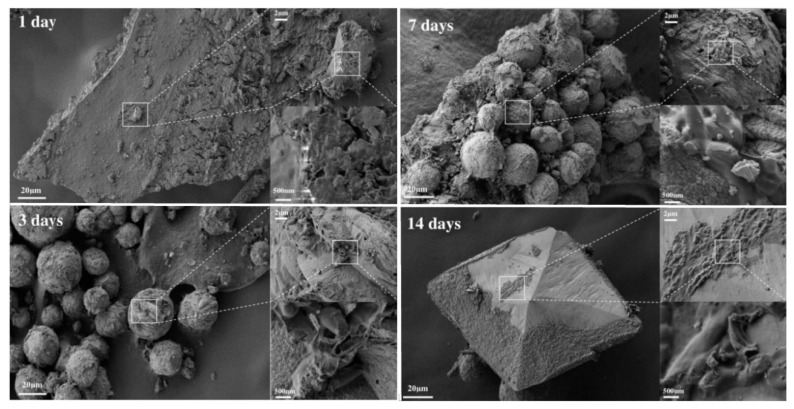
Representative scanning electron micrographs of CaCO_3_ precipitates appeared during 14-days of *B. licheniformis DSMZ* 8782 growth in B4-AC medium.

XRD and SEM analyses of samples taken from the liquid B4-U medium with growing strain allowed us to follow the CaCO_3_ biomineralization during 14 days. After the first day of the growth, we detected peaks characteristic to calcite and hardly observable peaks of vaterite (Figure 8, red line) followed by accumulation of both vaterite and calcite phases after 48 h (Figure 8, green, blue and violet lines). SEM analysis confirmed that the amorphous phase of calcium carbonate consisted of ultrasmall particles (10–20 µm) with irregular shape and was formed on the first day (Figure 9). Then the precipitates were transformed into ellipsoid or spherical particles (30–50 µm) characteristic to the polymorph vaterite; on the second day they increased in both size and quantity. Closer to the 14th day we observed appearance of large particles (80–120 µm) with well-defined faceting typical to calcite (Figure 9). Note that non-biogenic and biogenic processes of calcium carbonates crystallization are rather complex and proceed through various intermediates [17,38,40] depending on various factors like reaction and culture medium composition, bacteria species, aeration process and others [41,42], so that it can be considered typical for both bacterially induced vaterite or calcite microcrystals.

The ability of microorganisms to induce calcium carbonate precipitation through different mechanisms is well known. However, most reports on MICP processes describe only one of the mechanisms inducing microbial biomineralization of calcium carbonate [6,11,22,23,24,43] and others. For some bacteria (*Bacillus, Arthrobacter* and *Rhodococcus*) the induction of Ca carbonate crystallization in a medium containing calcium organic acid salts (acetate, lactate, citrate, succinate) was described [44].

Helmi with coauthors showed the induction of calcium carbonate precipitation in different urea-containing media during the growth of the bacterium *B. licheniformis* [45]. Barabesi et al. reported the relationship between the activity of cell genes responsible for the induction of the biogenic mineralization process and the metabolism of fatty acids [46]. Only the recent publication of Reeksting et al. described the ability of microorganisms to induce CaCO_3_ precipitation through two different mechanisms depending on the growth conditions [47]. The aforementioned authors showed that different polymorphs were formed with a dependence on different nutrients added into the growth medium and, accordingly, the process depended on the mechanism that led to the CaCO_3_ precipitation. Our results confirm that this phenomenon is more widespread than previously thought. All selected ureolytic bacteria retained their ability to induce CaCO_3_ mineralization in urea-deficient medium (Table 1 and Table 2) although the transformation of CaCO_3_ morphologies occurred at a slower rate than along the ureolytic pathway. Most likely, this behavior cannot be explained only by the species-specificity of bacteria or non-biogenic crystallization due to elevated content of Ca^2+^ and carbonate ions in the media that was verified in experiments without bacteria. We suppose that the explanation is in switching the genes coding enzymes (at least, carbonic anhydrases and ureases as was reported for various representatives of the genus *Bacillus* [26,27]) that catalyze reactions resulting in the formation of carbonate ions. Since all the ureolytic strains that we selected were capable of inducing CaCO_3_ precipitation regardless of the presence of urea in the medium, such flexibility of bacteria can be considered the rule rather than the exception. Thus, our findings make it possible to control the production of calcium carbonate polymorphs. It is important not only in view of MICP application in civil engineering for the restoration of building constructions, but for drug delivery, regenerative medicine and tissue engineering as bone cements and substitute materials, dental implants and scaffolds as well as for the design of new materials reviewed in [18,48].

In conclusion, for each of the selected and characterized bacterial candidates, the transformation “ACC-to-vaterite-to-calcite” occurs by different ways, yielding different amounts of precipitates and variable morphologies of carbonate minerals in media with and without urea. In urea-containing medium, all selected bacteria showed a high level of specific urease activity accompanied by the process of biomineralization. In the urea-free medium, all ureolytic bacteria also induced the CaCO_3_ precipitation, although at lower pH values. We found that only five strains (*B. licheniformis* DSMZ 8782, *B. cereus* 4b, *S. epidermidis* 4a, *M. luteus* BS52, *M. luteus* 6) were able to completely repair the original shape of the cement-sand samples. Detailed studies of the most potent strain *B. licheniformis* DSMZ 8782 revealed the slower rate of the polymorph transformation in the urea-deficient medium than in urea-containing one. This remarkable property could be used in various industrial applications where a specific polymorph (ACC, vaterite or calcite) is required.

## Figures and Tables

**Table 1:** CaCO_3_ precipitates produced during the bacteria growth in two media.

Microorganism	Quantity of Precipitates ^a^ Produced by Studied Strains in 100 mL of	
	B4-U Medium, mg ^b^	B4-AC Medium, mg ^b^
*B. licheniformis* DSMZ 8782	180.9 ± 8.1	113.0 ± 4.7
*S. epidermidis* 4a	234.5 ± 1.5	121.2 ± 3.8
*B. cereus* 4b	217.8 ± 12.7	110.3 ± 4.1
*M. luteus* 6	207.5 ± 8.3	187.6 ± 5.4
*B. subtilis* K51	120.2 ± 4.5	167.2 ± 9.3
*B. subtilis* 170	151.9 ± 2.8	170.6 ± 6.8
*B. cereus* 168	180.6 ± 5.9	120.0 ± 15.5
*M. luteus* BS52	157.7 ± 0.1	233.5 ± 12.9
*B. cereus* BSP	155.5 ± 5.4	220.6 ± 6.9
*E. coli* DH5α	0	0

^a^ Insoluble precipitates were thoroughly collected from the flask walls with a scraper, separated from the planktonic cells and the medium by filtration, washed with distilled water, oven dried at 50 °C, and weighed. ^b^ Errors bars represent average and standard deviation of three replicates.

**Table 2:** Results of powder X-ray diffraction (XRD) analysis of CaCO_3_ polymorphs formed by bacterial strains under study.

Microorganism	B4-U Medium			B4-AC Medium		
	Calcite	Vaterite	Unidentified Phase	Calcite	Vaterite	Unidentified Phase
*B. licheniformis* DSMZ 8782	+	-	-	+	-	+
*S. epidermidis* 4a	-	-	+	+	+	-
*B. cereus* 4b	-	-	+	+	+	-
*M. luteus* 6	+	-	+	+	+	+
*B. subtilis* K51	+	-	-	+	-	+
*B. subtilis* 170	+	+	-	+	+	+
*B. cereus* 168	+	+	-	+	+	+
*M. luteus* BS52	-	-	+	-	+	+
*B. cereus* BSP	+	-	-	+	+	+

## Supplementary Materials

The following are available online at https://www.mdpi.com/2075-1729/10/12/317/s1, Figure S1: Typical x-ray diffraction patterns of CaCO_3_ precipitates formed by representative bacterial strains under study: (a) *B. subtilis* K51 after the growth in B4-U (calcite), (b) *B. cereus* 4b after the growth in B4-AC (calcite+vaterite), (c) *M. luteus* 6 after the growth in B4-AC (calcite + vaterite + non-identified phase). Figure S2: Representative scanning electron micrograhs of CaCO_3_ precipitates formed by some bacterial strains under study: (a) vaterite (ellipsoid particles) produced by *S. epidermidis* 4a in B4-AC medium; (b) calcite, *B. licheniformis* DSMZ 8782, B4-U medium; (c) vaterite, *B. subtilis* 170, B4-AC medium. Bacterial cells and imprints are seen on the surface of the crystal; (d) calcite (large faceted crystal) and vaterite (ellipsoid particles), *B. subtilis* 170, B4-AC medium. Figure S3: Typical images of micro-crack filling by *B. licheniformis* DSMZ 8782 before (a and c) and after a month of the growth on the cement surface in B4-U medium (a and b) and in B4-AC medium (c and d). Cement samples were 4.5 × 1 cm in size; microcracks were 3.5–4 cm in length and 0.12–2.0 mm in width).
