# Supplementary material for: Calcifying Bacteria Flexibility in Induction of CaCO3 Mineralization
Source: Life (Basel). 2020 Nov 28;10(12):317. doi: 10.3390/life10120317 (PMC7759876; doi:10.3390/life10120317)
Supplement: Supplementary file 1 [file life-10-00317-s001.pdf]

life

MDPI

Supplemental materials

Calcifying Bacteria Flexibility in Induction of $CaCO_3$ Mineralization

Darya A. Golovkina[1,2], Elena V. Zhurishkina[1,2], Lyubov A. Ivanova[1,2], Alexander E. Baranchikov[3], Alexey Ye. Sokolov[1], Kirill S. Bobrov[1,2], Alexey E. Masharsky[4], Natalia V. Tsvigun[5], Gennady P. Kopitsa[1], Anna A. Kulminskaya[1,2*]

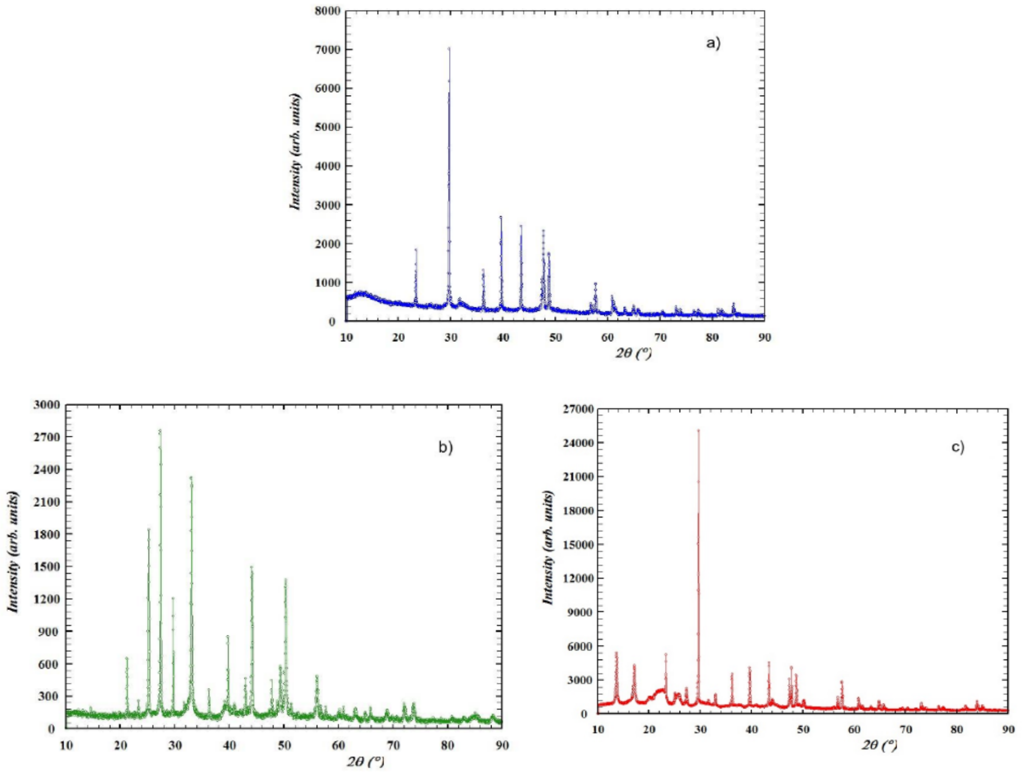

Figure S1. Typical x-ray diffraction patterns of $CaCO_3$ precipitates formed by representative bacterial strains under study: (**a**) *B. subtilis* K51 after the growth in B4-U (calcite), (**b**) *B. cereus* 4b after the growth in B4-AC (calcite + vaterite), (**c**) *M. luteus* 6 after the growth in B4-AC (calcite + vaterite + not identified phase).

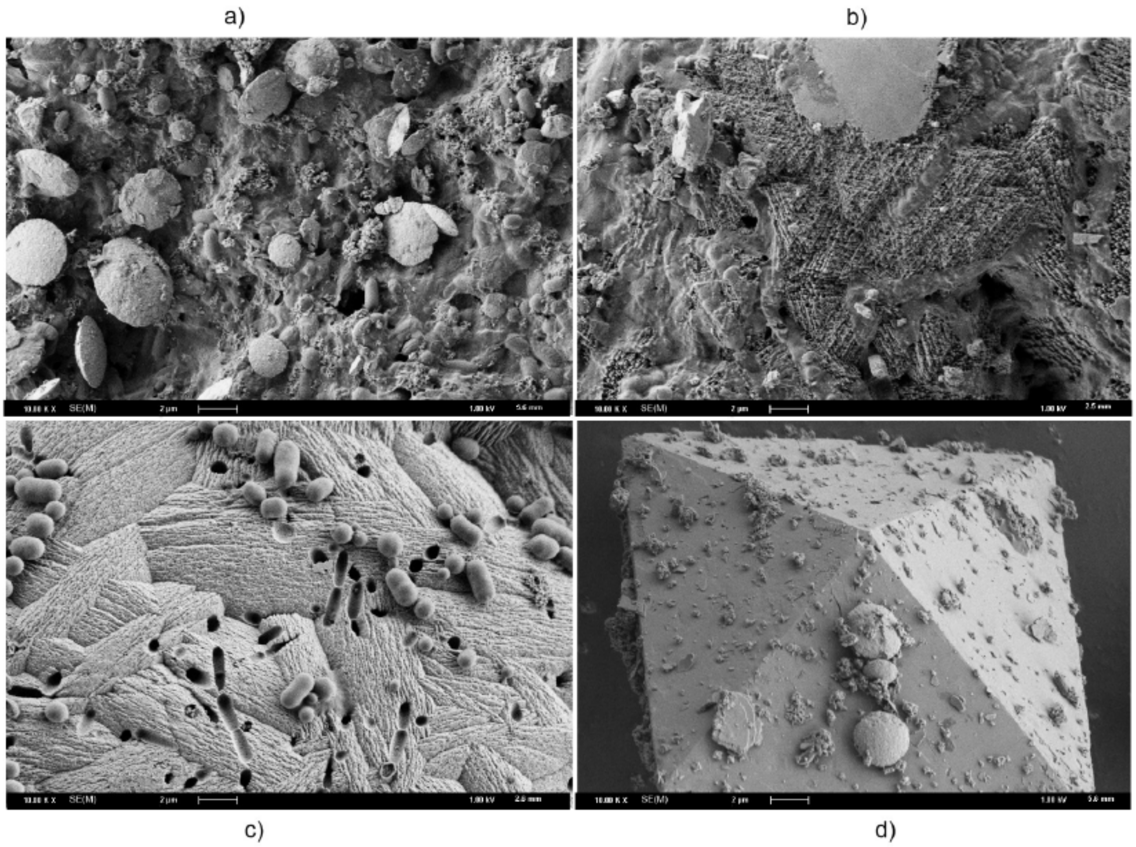

Figure S2. Representative scanning electron micrograhs of $CaCO_3$ precipitates formed by some bacterial strains under study: (**a**) vaterite (ellipsoid particles) produced by *S. epidermidis* 4a in B4-AC medium; (**b**) calcite, *B. licheniformis* DSMZ 8782, B4-U medium; (**c**) vaterite, *B. subtilis* 170, B4-AC medium. Bacterial cells and imprints are seen on the surface of the crystal; (**d**) calcite (large faceted crystal) and vaterite (ellipsoid particles), *B. subtilis* 170, B4-AC medium.

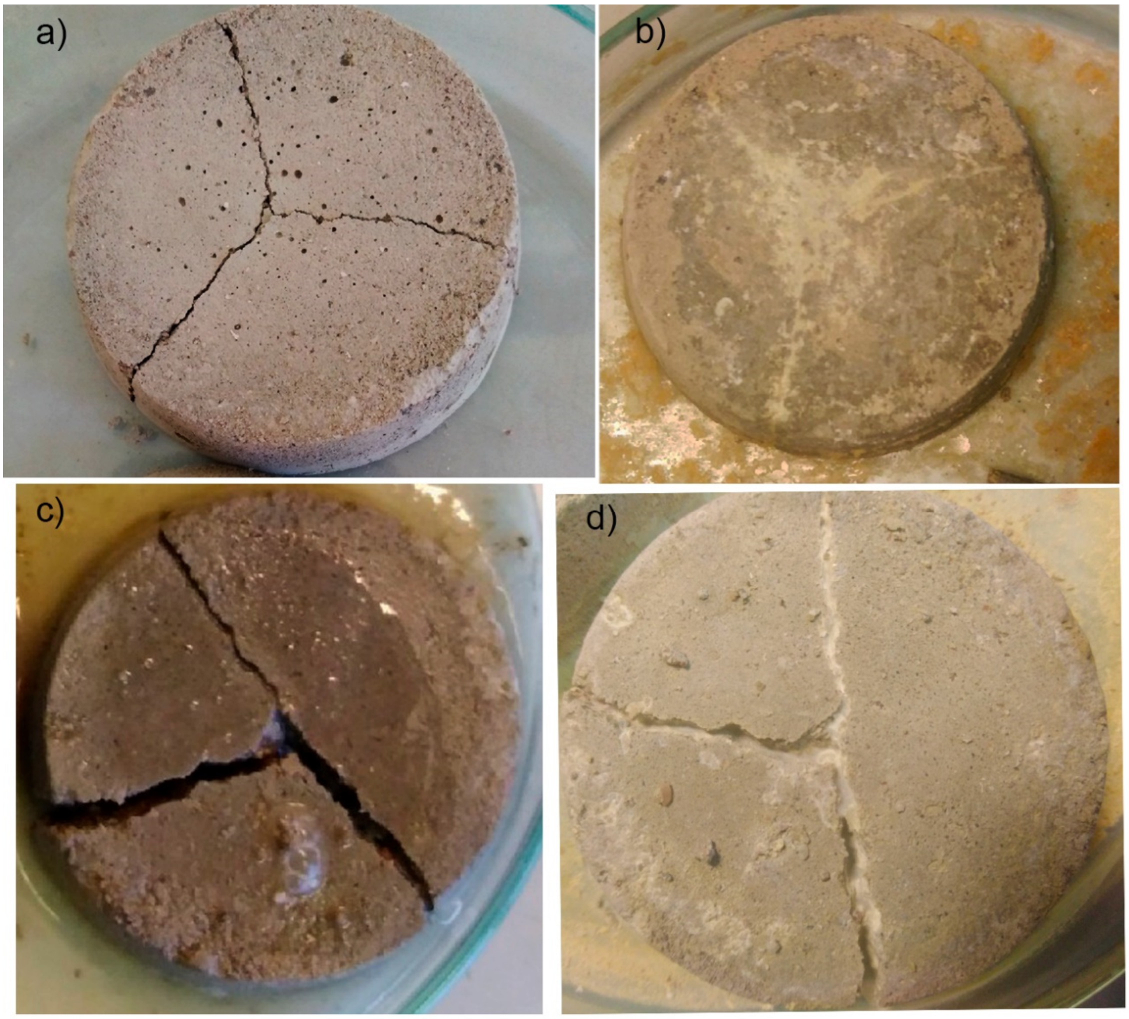

Figure S3. Typical micrographs of micro-crack filling by *B. licheniformis* DSMZ 8782 before (**a and c**) and after a month of the growth on the cement surface in B4-U medium (**a and b**) and in B4-AC medium (**c and d**).

Publisher's Note: MDPI stays neutral with regard to jurisdictional claims in published maps and institutional affiliations.

© 2020 by the authors. Licensee MDPI, Basel, Switzerland. This article is an open access article distributed under the terms and conditions of the Creative Commons Attribution (CC BY) license (http://creativecommons.org/licenses/by/4.0/).
